# Novel digital speech biomarker for early detection of Alzheimer’s disease

**DOI:** 10.1002/alz.083421

**Published:** 2025-01-03

**Authors:** Alexandra König, Nicklas Linz, Johannes Tröger

**Affiliations:** ^1^ ki elements UG, Saarbrücken Germany; ^2^ CoBTeK (Cognition Behaviour Technology) Research Lab, University Côte d’azur, Nice France; ^3^ Memory Clinic and Research Centre, University Hospital, Nice France; ^4^ ki:elements GmbH, Saarbrücken Germany

## Abstract

Future clinical trials targeting Alzheimer’s disease (AD) on new disease modifying drugs necessitate a paradigm shift towards early identification of individuals at risk. Emerging evidence indicates that subtle alterations in language and speech characteristics may manifest concurrently with the progression of neurodegenerative disorders like AD. These changes manifest as discernible variations, assessable through semantic nuances, word choices, sentiment, grammar usage (linguistic features), and phonetic/acoustic traits (paralinguistic features). Consequently, automated analysis of speech performance stands as a promising avenue for detecting AD, enabling widespread screening of diverse at‐risk populations.

The talk will outline applications of novel digital speech biomarkers for measuring cognition (SB‐C) alongside its analytical and clinical validation. The SB‐C algorithm demonstrates robustness in detecting Mild Cognitive Impairment (MCI) across various cohorts and languages. In addition, speech and language markers have shown to be useful to assess objectively common neuropsychiatric symptoms in MCI such as depression or apathy.

This innovation holds the potential to enhance the efficiency of ongoing trials and augment future primary healthcare practices concerning AD. Identifying subtle cognitive and as well as affective changes through speech analysis signifies a critical advancement in the pursuit of early AD detection, potentially transforming the landscape of AD research and clinical interventions.

